# Immunological Insights into Opioid-Free Anaesthesia in Oncological Surgery: A Scoping Review

**DOI:** 10.1007/s11912-022-01300-5

**Published:** 2022-05-28

**Authors:** Laura Smith, Juan P. Cata, Patrice Forget

**Affiliations:** 1 School of Medicine, Medical Sciences and Nutrition, University of Aberdeen, Aberdeen, UK; 2 Department of Anesthesiology and Perioperative Medicine, The University of Texas MD Anderson Cancer Center, Houston, TX USA; 3 Anaesthesiology and Surgical Oncology Research Group, Houston, TX USA; 4 Institute of Applied Health Sciences, Epidemiology Group, School of Medicine, Medical Sciences and Nutrition, University of Aberdeen, Aberdeen, UK; 5 Department of Anaesthesia, NHS Grampian, Aberdeen, UK

**Keywords:** Opioid-free anaesthesia, Cancer, Immune response, Surgery

## Abstract

**Purpose of Review:**

The influence of opioids on outcomes after cancer surgery when used, or avoided, intraoperatively remains unclear. There is a need to conduct a scoping review to explore the wider context and provide direction for future research. The review will examine the current state of evidence in humans, with a focus on immunological biomarkers and clinically relevant cancer outcomes in trials comparing opioid-free to opioid-based general anaesthesia.

**Recent Findings:**

There is limited research on this subject area, which is mainly focused on breast cancer. The most frequently evaluated immunological parameter is the neutrophil-to-lymphocyte ratio. Cancer outcomes are mainly focused on recurrence.

**Summary:**

The central knowledge gap is understanding how the cellular effects of opioids translate into longer-term patient outcomes. The major challenge for future research is accounting for the immunomodulatory effects of a wide range of confounding factors, which have yet to be clarified.

**Supplementary Information:**

The online version contains supplementary material available at 10.1007/s11912-022-01300-5.

## Introduction

The growing public health burden of cancer is placing enormous demands on health services that are already stretched. By 2040, it is expected that there will be 28.4 million cases of cancer: a 47% rise in the number of cases since 2020 [1]. Despite success rates of surgical intervention, cancer recurrence after surgery remains a recognised challenge with significant implications on prognosis and quality of life [2]. Many hypotheses have been made about the reasons why the perioperative period could contribute to cancer progression. There is growing interest in the effects of opioids used during the intraoperative and early postoperative period on the immune system, as their immunomodulatory effects may influence cancer recurrence after surgery [3].

Literature has highlighted for decades that opioids can affect a wide range of immune cells of both the innate and adaptive immune systems and inflammatory mediators such as cytokines [4, 5]. Effects are variable between opioids and seem to be dose-dependent [6]. Inflammation is not only a response to tumour cell invasion, but the process itself drives all stages of carcinogenesis [3]. The burden of a tumour reflects the relative balance between the strength of the suppressive immune response and the ability of the tumour cells to escape or inhibit immunosurveillance [7]. Whilst much of the evidence is based on experimental animal models, the described immunomodulatory effects are clearly of potential significance in the context of cancer surgery, which raises interesting questions about whether opioids should be avoided in the intraoperative period.

Studies have reported weak associations between intraoperative opioid exposure and survival end points [8, 9]. A recent study reported a protective association between intraoperative opioid use and recurrence-free survival in triple negative breast cancer [10]. Conclusions of systematic reviews evaluating the effects of opioids in patients with cancer have a common theme: there is a lack of robust evidence and high-quality trials in humans [11, 12, 13•]. Evaluation of the association between intraoperative opioid use and clinically relevant cancer outcomes is crucial to gain insight into how the cellular effects may affect longer-term outcomes, such as survival endpoints.

Linked with oncological outcomes is the concept of immunological biomarkers in cancer surgery: an area of growing interest in literature. The neutrophil-to-lymphocyte ratio, for example, has been reported as a useful prognostic marker in a range of cancers [14, 15]. Opioid-free anaesthesia (OFA) uses multimodal analgesia. Commonly used pharmacological agents include ketamine, lidocaine, and magnesium sulphate. OFA is often complemented by the use of loco-regional techniques to enhance analgesia [16•]. Immunological biomarkers may therefore be useful to identify subgroups of patients who would benefit most from OFA regimes in cancer surgery.

To provide direction for future research and ultimately inform anaesthetic practice, there is a major need to undertake a scoping review to examine the current evidence on the immunological aspects of opioid-free anaesthesia in adults undergoing cancer surgery and identify knowledge gaps. Unlike systematic reviews, scoping reviews do not aim to answer precise questions relating to specific outcomes but rather to map the current evidence in a systematic way to explore the wider context. Scoping reviews form a crucial preliminary step for research development and planning [17]. In the context of the immunological aspects of OFA, no such scoping review exists in current literature. This review, therefore, aims to address this important gap.

## Methods

### Overview

A scoping review was conducted between January 2021 and February 2021. The Preferred Reporting Items for Systematic reviews and Meta-Analyses extension for Scoping Reviews (PRISMA-ScR) guidelines were followed [18]. A protocol for the scoping review was firstly developed, comprising of the following steps: exploratory investigation of the literature; identification of the research aims; formulation of eligibility criteria; database search; screening and identification of studies; and extraction and charting of data.

### Initial Search

An exploratory investigation of the literature was undertaken with unrestricted criteria across a variety of databases to gain a broad overview of the topic area. This investigation guided both the selection of keywords for the final search and the formulation of the eligibility criteria.

### Search Strategy and Screening

A search was then conducted across five electronic databases: Cochrane Central Register of Controlled Trials (CENTRAL), MEDLINE/PubMed, Embase, Web of Science and clinicaltrials.gov. The most recent search was undertaken on February 9, 2022. Keywords included “opioid-free”, “anaesthesia” and “cancer surgery” and an extensive range of immunological keywords such as “neutrophil-to-lymphocyte ratio”, “cytokines” and “immune response”. Table S1 in Appendix A presents the full range of keywords used in the search processes. Senior librarian information assistants were consulted to maximise the comprehensive scope of the search. Detailed individual search strategies for each database are presented in Appendix B.

Following identification of 26 studies, references of these articles were scanned to identify any relevant studies not captured by the search. No additional articles for inclusion were identified from the reference lists. Duplicates were removed, leaving 15 studies. Articles were carefully screened against the predefined eligibility criteria by title and abstract (or study description if an ongoing clinical trial) then full-text.

### Eligibility Criteria

Studies assessing opioid-free general anaesthesia in adults undergoing cancer surgery were included, specifically randomised controlled trials in humans. Studies which did not assess any immunological parameters were excluded. Ongoing randomised trials were included to obtain insight into the remaining scope of research within this field. There was no restriction on data range to maximise the comprehensive nature of the search. Studies published in a non-English language were excluded.

The screening process was performed in duplicate. A total of eight articles were identified for final inclusion.

### Data Extraction and Charting

#### Published Randomised Controlled Trials

Study characteristics: type of cancer, type of surgical procedure, population age range eligibility, study population size

Anaesthesia regimes: pharmacological agents used for induction and maintenance of anaesthesia, use of loco-regional analgesia

Outcomes: immunological parameters and clinically relevant cancer outcomes with timepoint of evaluation

Summary of findings: main immunological or oncological findings

Additional study characteristics (Appendix C): age of participants, body mass index, gender, tumour location (Table S6a); smoking status, duration of operation (Table S6b)

### Clinical Trials

Study characteristics: trial status, type of cancer, type of surgical procedure, estimated number of participants

Anaesthesia regimes: pharmacological agents used for induction and maintenance of anaesthesia, use of loco-regional analgesia

Outcomes: immunological parameters and clinically relevant cancer outcomes with timepoint of evaluation.

### Synthesis of Results

In line with scoping review methodology, a description of the results is provided without formal data analysis to map the current state of evidence rather than address precise clinical questions on the impact of OFA in cancer surgery on specific outcomes.

## Results

### Study Retrieval Summary

The search strategy identified 26 studies, of which 11 were duplicates, leaving 15 studies. Following screening of titles and abstracts or study descriptions, nine studies remained. One study was excluded after full-text analysis, leaving eight studies for final inclusion. A common reason for exclusion applied to all seven excluded studies: the studies did not assess immunological outcomes. Figure S1 in Appendix A presents a flow diagram of the literature retrieval process, adapted from the PRISMA flow diagram [19].

### Individual Database Searches

#### Cochrane Central Register of Controlled Trials (CENTRAL)

A search conducted on the Cochrane Central Register of Controlled Trials (CENTRAL) in February 2021 yielded nine trials. Two articles were published randomised controlled trials. Of the nine trials, six were identified as eligible for inclusion. Two were published randomised controlled trials, three were clinical trials with a status of ‘recruiting’, and the remaining trial was of unknown recruitment status.

#### Embase

A search of Embase identified five randomised controlled trials, of which one, a published trial, was deemed eligible for inclusion.

#### MEDLINE/PubMed

A search of MEDLINE/PubMed identified two published randomised controlled trials. Both were duplicates of previously identified articles.

#### Web of Science

A search using the keywords “opioid-free”, “anaesthesia” and “cancer surgery” yielded two published randomised controlled trials. One was excluded as a duplicate, and the other excluded at full-text level.

#### Clinicaltrials.gov

A search of clinicaltrials.gov was conducted to identify studies not captured by the search of CENTRAL. Eight clinical trials were identified using the keywords “opioid-free anaesthesia”, “surgery” and “cancer”. One trial was deemed eligible for inclusion.

### Study Characteristics

#### Published Randomised Controlled Trials

Tables S2a and b in Appendix A present the study characteristics and anaesthesia regimes, respectively. Two studies included patients with prostate cancer and breast cancer respectively. Titon et al. included patients undergoing resection of a broad range of tumours. In this study, no information was provided about whether the surgery was open or laparoscopic. Furthermore, the benign or malignant nature of the tumours was not specified. The population size in the study by Rangel et al. was over three times larger than that of both remaining studies with a total of 143 patients. In the study by Rangel et al., only patients with a moderate to high risk of biochemical recurrence were included.

In relation to anaesthetic techniques, in the study by Aboalsoud et al., anaesthesia was maintained with an inhalational agent (isoflurane), whereas the other studies used intravenous agents alone. Loco-regional analgesia was used to complement the OFA regime in the studies by Rangel et al. and Titon et al. In the study by Rangel et al., two patients in the OFA group required fentanyl due to presumed failure of the loco-regional block.

Lidocaine was used for induction and maintenance of anaesthesia in two of the three studies. Rangel et al. used an NMDA antagonist, dextroketamine, and Titon et al. used magnesium sulphate as part of the anaesthetic regime.

#### Ongoing Clinical Trials

Tables S3a and b in Appendix A present the study characteristics and anaesthesia regimes, respectively. Of the five trials, four have a status of ‘recruiting’. One study has an unknown recruitment status, which is defined by clinicaltrials.gov as no verification for more than 2 years past the study completion date. Three of five studies are evaluating OFA in breast cancer. The second most common cancer to be evaluated is non-small cell lung cancer. The estimates for study population sizes are similar across studies.

There is significant variability in the level of detail provided about the anaesthetic regimes. Loco-regional anaesthesia techniques are used in all studies. Only one study describes the use of an inhalational agent (desflurane); however, not all study descriptions are complete. One ongoing trial includes fentanyl in the OFA group if required for haemodynamic stability.

### Immunological Outcomes

#### Published Randomised Controlled Trials

Table S4 in Appendix A presents a description of the immunological parameters assessed across all studies included in this review. Table 1 presents the immunological and oncological outcomes assessed in published randomised controlled trials. Table S5 in Appendix A presents the summary of findings for the published randomised controlled trials to supplement the context of the review. Table 2 presents the outcomes assessed in ongoing clinical trials.

**Table 1:** Outcomes evaluated in published randomised controlled trials

	Immunological		Oncological	
Authors (year)	Biomarker	Timepoints of measurement	Parameter	Timepoint of measurement
Rangel et al. (2021)	Neutrophil-to-lymphocyte ratio	Preoperatively Postoperatively	Biochemical recurrence (*defined as prostate-specific antigen level > 0.2 ng/ml at 6 to 13 weeks after surgery*) Biochemical recurrence-free survival	PSA level every 6 months for 2 years after surgery
Aboalsoud et al. (2021)	IL-10 TNF-α Caspase 3	Preoperatively 24 h postoperatively 7 days postoperatively	N/A	N/A
Titon et al. (2021)	IL-4 IL-12 IL-17A TNF-α Oxidative stress profile (lipid peroxidation status and antioxidant capacity of plasma)	Preoperatively (in operating room) Immediate postoperative period (in operating room)	N/A	N/A

**Table 2:** Outcomes evaluated in ongoing randomised controlled trials

	Immunological			Oncological	
	Biomarker		Timepoint of measurement	Parameter	Timepoint of measurement
**Trial 1** Breast cancer	N/A		N/A	Cancer recurrence and metastases	Up to 12 months after surgery
**Trial 2** Breast cancer	Neutrophil-to-lymphocyte ratio Platelet-to-lymphocyte ratio Number of NK cells, T helper cells, cytotoxic T cells		1 day preoperatively Immediate postoperative period 24 h postoperatively	N/A	N/A
**Trial 3** Non-small cell lung cancer	Neutrophil-to-lymphocyte ratio Platelet-to-lymphocyte ratio Lymphocyte-to-monocyte ratio Advanced lung cancer inflammation index Systemic immune inflammation index	IL-6, IL-8, IL-10, TNF-α CRP WBC AVP (vasopressin) Cortisol HIF-1α VEGF NF-κB	Preoperatively End of surgery 24 h postoperatively	N/A	N/A
**Trial 4** Non-small cell lung cancer	N/A		N/A	Relapse-free survival Overall survival	Within 5 years
**Trial 5** Breast cancer	Neutrophil-to-lymphocyte ratio variation Primary: between preoperative period and 24 h postoperatively Secondary: between 1 and 24 h postoperatively		Preoperatively 1 h postoperatively 24 h postoperatively	N/A	N/A

All three published randomised controlled trials included in this review assessed immunological parameters. The most frequently evaluated parameter was the neutrophil-to-lymphocyte ratio. In the study by Rangel et al., the exact timepoints of measurement in the preoperative and postoperative periods were not specified. Postoperative neutrophil-to-lymphocyte ratio median values were not statistically different between groups, and no association was found between preoperative neutrophil-to-lymphocyte ratios and biochemical recurrence-free survival.

Aboalsoud et al. evaluated cytokine interleukin (IL)-10, tumour necrosis factor (TNF)-α and a marker of apoptosis, caspase 3, specifying the postoperative measurements as 24 h and 7 days after surgery. Statistically significant increases in IL-10 and caspase 3 were found in the opioid-free anaesthesia group compared to the opioid-based anaesthesia group.

Titon et al. evaluated the oxidative stress profile in addition to pro-inflammatory and anti-inflammatory cytokines. A statistically significant reduction in the postoperative lipid peroxidation was reported in the opioid-free anaesthesia group compared to the opioid-based anaesthesia group, but no significant differences in cytokine levels between the groups were found.

### Ongoing Clinical Trials

Three of five trials are evaluating immunological parameters. All studies report evaluation of the parameters preoperatively and 24 h postoperatively. Two trials specify additional time points of measurement: immediately after surgery and 1 h after surgery, respectively. Only one study specifies the timepoint of measurement in the preoperative period as 1 day before surgery.

### Oncological Outcomes

#### Published Randomised Controlled Trials

Only one study included in this review evaluated a clinically relevant cancer outcome. The study was conducted by Rangel et al. No statistically significant differences in biochemical recurrence or recurrence-free survival were reported between groups. Furthermore, no association was found between preoperative neutrophil-to-lymphocyte values and biochemical recurrence of prostate cancer.

#### Ongoing Clinical Trials

Of the five trials included in this review, only two report evaluation of clinically relevant cancer outcomes in the study description. Survival endpoints at 5 years are being evaluated by one study, specifically relapse-free survival and overall survival. No clarification was provided on the definition of relapse-free survival. The study in breast cancer reports evaluating cancer recurrence and metastases within a 12-month period, diagnosed by a breast cancer specialist.

## Discussion

### Overview of Evidence

This scoping review found that there are limited randomised controlled trials evaluating the immunological aspects of OFA in adults undergoing cancer surgery. The published trials included in this review have been conducted recently, highlighting that this is an area of growing interest and a research priority. Most of the evidence is in the context of breast cancer surgery. In 2020, the highest number of new cancer cases was attributed to breast cancer, and surgery is the most common treatment intervention for this type of cancer [20, 21]. Thus, there is clear justification for the evaluation of opioid-free anaesthesia in this patient population. A wide range of immunological parameters are being assessed, and cancer outcomes are focused on recurrence.

### Immunological Parameters

The neutrophil-to-lymphocyte ratio, the most frequently assessed immunological parameter in this review, has been reported to be a useful prognostic marker in a range of cancers, although a recent umbrella review reported a lack of robust evidence to support the generalisability of its use as a biomarker [22•]. Traditionally, neutrophils were considered to have anti-tumour activity; it is now recognised that neutrophils have more diverse and opposing roles: functional plasticity exists. Neutrophils can have pro-tumour or anti-tumour activity depending on the tumour microenvironment. The tumour microenvironment refers to the diverse collection of stromal cells, immune cells and inflammatory mediators surrounding the tumour and is a dynamic environment [23••, 24]. Considering the influence of the tumour microenvironment on neutrophil activity, the neutrophil function may be more important than a ratio in understanding how these parameters may be surrogate markers for survival endpoints. This concept could apply to other cellular parameters assessed such as the lymphocyte-to-monocyte ratio.

Accumulating evidence on the role of the tumour microenvironment has stimulated interest in its evaluation through scoring systems. Specific immune cell populations can be measured from resected tissue samples and analysed using image techniques to form a score, which can be used as a prognostic indicator. The National Institute of Health and Care Excellence (NICE) supports the use of Immunoscore for localised colorectal cancer for predicting response to immunotherapy, survival endpoints and prognosis of patients with metastatic disease [25]. Immune scores obtained from staging biopsies may be more useful than cellular biomarkers as opioids have different effects on different cells. Knowledge of the relative proportions of cellular subpopulations may therefore provide a more accurate insight into the likely impact and significance of intraoperative opioid administration for different patients. At a cost of £2,250 per test, there are clear financial limitations. The cost-effectiveness may be supported by potential long-term benefits associated with the prevention of relapse, but at present, there is a lack of robust evidence. Currently, the neutrophil-to-lymphocyte ratio represents a pragmatic choice for evaluation as the ratio is easily obtainable from the full blood count. If the use of tumour microenvironment scores is not justified per se, then these scores could be used alongside more traditional cellular biomarkers. These scores may help to tailor the measurement of certain biomarkers to specific patient populations, as well as guide the identification of threshold values.

In addition to prognostic value, another important consideration is the timepoint of measurement of biomarkers. In this review, most postoperative measurements across the parameters assessed were in the immediate postoperative period and 24 h after surgery. It is recognised that within hours of surgery, a pro-inflammatory response occurs, followed by a longer compensatory immunosuppressive state which can last for up to 2 weeks [26••]. Thus, to understand the impact of opioid-free anaesthesia more fully, the parameters should ideally be evaluated over a longer postoperative period.

#### Other Parameters

One ongoing trial is evaluating markers more directly related to tumour cell activity: caspase-3 (a marker of apoptosis), hypoxia-inducible factor 1-alpha and NF-κB (transcription factors associated with cell proliferation) and VEGF (a pro-angiogenic growth factor).

The evidence on cellular proliferation, migration and angiogenesis is contentious. Conflicting pro-tumour and anti-tumour effects have been reported, but there is significant heterogeneity amongst studies [27, 28, 29]. This picture is further complicated by evidence to suggest that expression of the mu-opioid receptor in the absence of exogenous opioids promotes activation of signalling proteins, specifically Akt and mTOR kinases. Activation of these signalling proteins is associated with cancer progression [30]. In this context, it would therefore be important to evaluate differences in mu-opioid receptor expression levels between tumours. Thus, there are evidently many confounding factors limiting the use of this group of parameters as biomarkers.

### Clinically Relevant Cancer Outcomes

The clinically relevant cancer outcomes assessed in the included studies were mainly focused on cancer recurrence. The term ‘recurrence’ requires clarification; however, as the term encompasses different circumstances. The National Cancer Institute describes two possible interpretations of the term. Firstly, recurrence of cancer from residual tumour cells. Secondly, a more generic use of ‘recurrence’: new occurrence of cancer arising from cells that are biologically independent of the original tumour [31]. It is important to distinguish between these interpretations. If there is a higher incidence of cancer arising from residual cells, this may point towards site-specific immune effects, whereas a higher incidence of new occurrences may place more weight on a more global immunosuppressive state induced by opioids.

Survival endpoints are commonly evaluated in the context of cancer; however, a systematic review conducted by Shrestha et al. highlighted that quality of life may be more important to some patients with cancer than length of life [32]. This is a key consideration for future research.

### Confounding Factors

#### Anaesthetic Agents and Techniques

There is ongoing discussion about the effects of anaesthetic agents on the immune system and cancer cells, particularly relating to volatile agents, described in the anaesthesia regimes of two studies. Pro-tumour and anti-tumour effects of inhalational anaesthetics have been reported across a range of primarily in vitro studies, which differ widely across a range of study characteristics including type of cancer, volatile agent and concentration and duration of anaesthetic exposure [33, 34, 35]. Furthermore, it is well-established that loco-regional analgesia techniques reduce the surgical stress response compared to general anaesthesia, but the impact on cancer recurrence appears to be minimal based on a recent randomised controlled trial [36].

#### Opioid-Sparing Pharmacological Agents

Lidocaine has recognised anti-inflammatory properties, particularly in terms of lymphocyte proliferation and cytokine production [37]. There is recent evidence that lidocaine also has direct effects on cancer progression through alteration of cell signalling pathways leading to reduced cellular proliferation and induction of apoptosis; however, most of the evidence is based on in vitro models [38, 39]. There is currently very limited evidence on the impact of intraoperative intravenous lidocaine on clinical outcomes in the context of cancer and a clear need for high-quality randomised controlled trials [40]. The immunological effects of ketamine are less well described, but the evidence suggests that the immunomodulatory effects may lie in an immunosuppressive direction, even if it may be model-dependent [41, 42]. Whilst there is evidence to suggest that intraoperative ketamine exposure is associated with improved recurrence outcomes, the heterogeneity amongst studies with associated confounding factors limits overall conclusions about the long-term effects of intraoperative ketamine [43, 44]. Thus, as the significance of the immunomodulatory effects of opioid-sparing pharmacological agents remains uncertain, at present, the evidence is not robust enough to justify their avoidance.

#### Patient Demographics and Surgical Intervention

Future research on OFA in cancer surgery will benefit from consistent reporting of patient demographics, specifically smoking, older age and increased body mass index. These variables all have reported immunomodulatory effects [45, 46]. Additional study characteristics for the published randomised controlled trials are presented in Tables S6a and b in Appendix C. Only one of three published trials included in this review reported on the smoking status of study participants. Smoking is associated with a range of immunological effects including changes in the relative proportions of T lymphocyte populations [46].

Most of the patients in the studies included in this review underwent open surgery. Laparoscopic surgery is associated with a reduced pro-inflammatory response compared to open surgery, even if a recent study did not identify any differences in terms of clinical outcome [47, 48]. Furthermore, the duration of surgery is also important due to prolonged physiological stress and a potentially longer duration of opioid use. The study by Rangel et al. did not report on operative duration.

#### Nociception, Pain and Inflammation

Pro-inflammatory cytokines such as IL-6 and TNF-α, both parameters assessed by studies in this review, are associated with nociceptive activation [49]. Exploration of the impact of nociceptive activation and pain on immunological parameters in the context cancer surgery was out with the scope of this review; however, this is an interesting opportunity for future research.

#### Immunotherapy

There is growing interest in combination regimes of neoadjuvant immunotherapy and surgery to improve postoperative cancer outcomes. Immunotherapy refers to treatments, such as monoclonal antibodies, which modulate immune responses to influence disease progression. In the context of opioid use, there is evidence to suggest concomitant opioid use alongside immunotherapeutic agents is associated with cancer progression [50]. This also raises interesting questions about the optimal timing for surgery after immunotherapy. It is possible that in the long-term, advances in immunotherapy may reduce the need for surgical intervention, but at present, there is a need for further evaluation of this area.

### Limitations of Review

A limitation of this review is the lack of generalisability of the findings, owing to significant heterogeneity amongst studies. A critical appraisal of the evidence was not conducted, owing to the nature of a scoping review; therefore, comments cannot be made on the quality of the evidence. Furthermore, in the study by Titon et al., patients were included if they were undergoing resection of a tumour. As the characteristics of the malignancies were not defined, some of tumours may have been benign, thus decreasing the generalisability of findings.

### Summary of Current evidence, Knowledge Gaps and Future Research Priorities

**Fig. 1:**
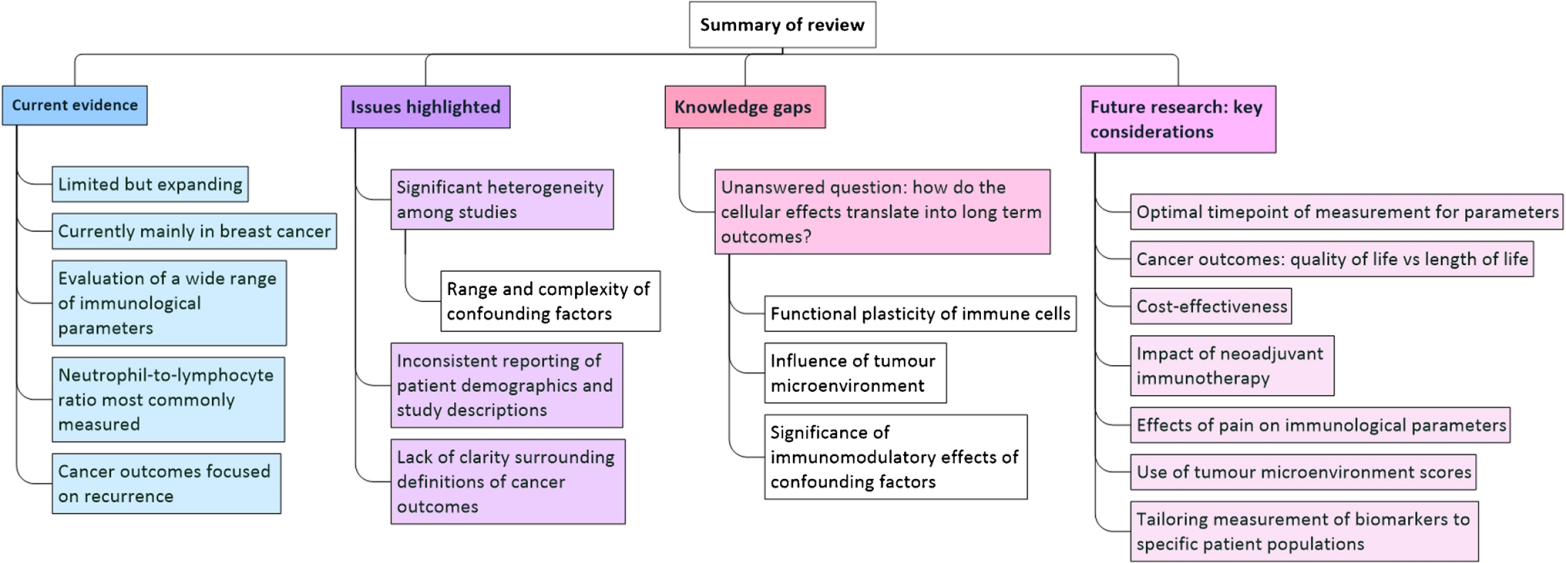
Summary of review (created using MindManager®)

Figure 1 presents a summary of the current state of evidence, knowledge gaps and key considerations for future research.

## Conclusions

This scoping review provides a systematic overview of the evidence on the immunological aspects of OFA in adults undergoing cancer surgery, with a specific focus on immunological biomarkers and clinically relevant cancer outcomes. It has highlighted the extent of knowledge gaps and considerations for future research including consistent reporting of data and definitions. It is clear there is a strive towards understanding the association between the cellular effects of opioids and prognostic outcomes, but the major challenge in evaluating the potential benefits of OFA is accounting for the multitude of confounding factors which have immunomodulatory effects yet to be clarified. Considering these complexities, the goal of informing anaesthetic practice may be a long way off, but optimism lies within the growing body of research and interest in this field, paralleled with advances in clinical medicine. These are small but significant steps in an area to follow with great interest.

## Supplementary information

ESM 1 (DOCX 200 kb)
