# Supplementary material for: Immunological Insights into Opioid-Free Anaesthesia in Oncological Surgery: A Scoping Review
Source: Curr Oncol Rep. 2022 May 28;24(10):1327–36. doi: 10.1007/s11912-022-01300-5 (PMC9474513; doi:10.1007/s11912-022-01300-5)
Supplement: Supplementary file 1 — (DOCX 200 kb) [file 11912_2022_1300_MOESM1_ESM.docx]

**Appendices**

*Appendix A*

| **Core** | **Immunological** | **Oncological** |
| --- | --- | --- |
| Opioid-free | Inflammation | Metastases |
| Anaesthesia | Immune system | Recurrence |
| Cancer surgery | Immune response | Angiogenesis |
|  | Neutrophil-to-lymphocyte ratio | Tumour growth |
|  | Platelet-to-lymphocyte ratio | Recurrence-free survival |
|  | Cytokines | Overall survival |
|  | Natural killer cells (NK) | Apoptosis |
|  | Neutrophils |  |
|  | Lymphocyte |  |

***Table S1*** *Range of keywords used for the database searches*

**
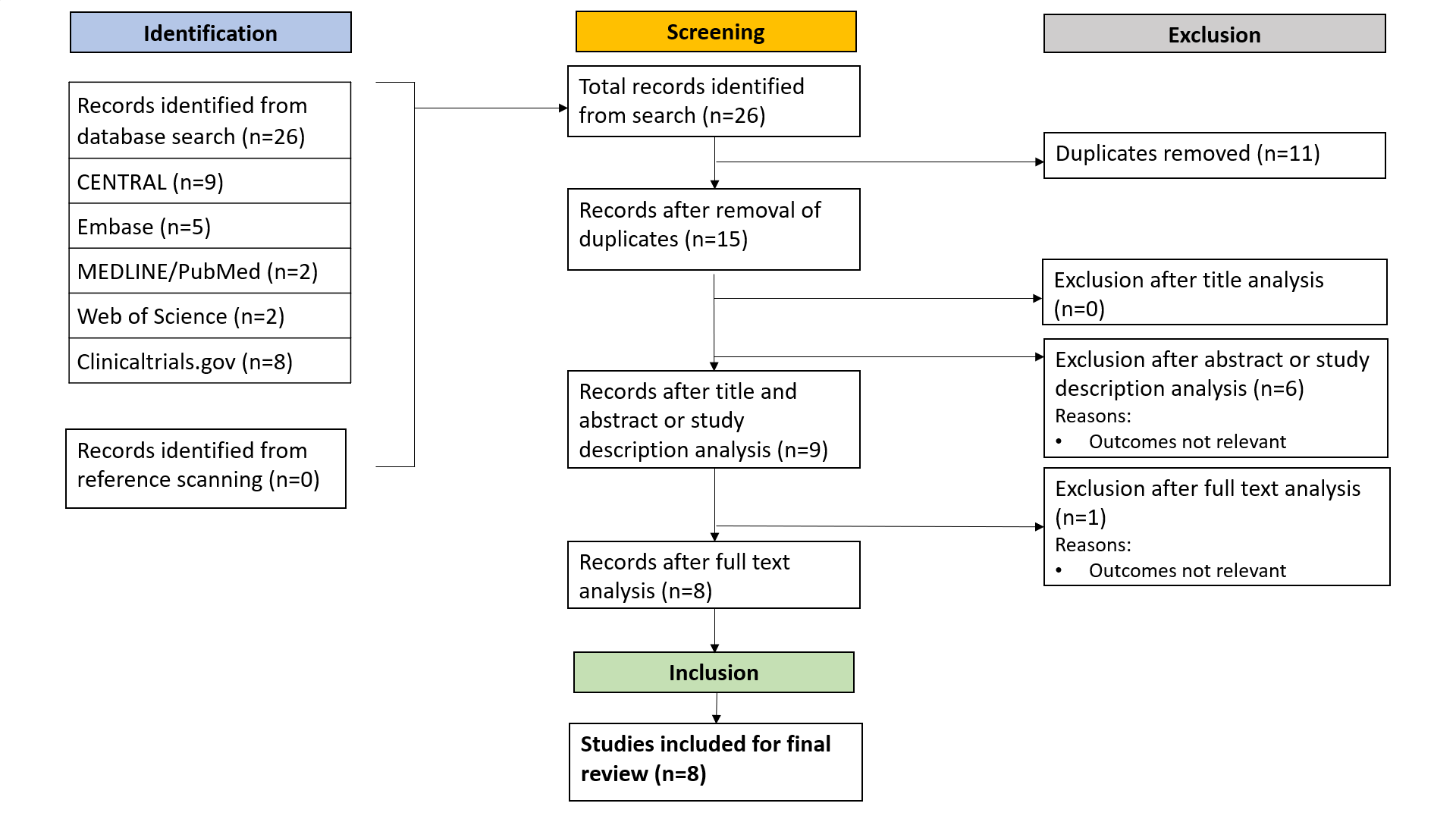
**

***Fig S1*** *Flow diagram illustrating the process of study identification, screening and identification of studies for inclusion. Adapted from PRISMA flow diagram.*[19]

|  |  |  |  | **Population size** | | |
| --- | --- | --- | --- | --- | --- | --- |
| **Authors (Year)** | **Type of cancer** | **Surgical procedure** | **Population age eligibility** | **Opioid-free anaesthesia** | **Opioid-based anaesthesia** | **Total** |
| Rangel *et al* (2021) | Prostate cancer (localised with moderate or high risk of biochemical recurrence according to D'Amico criteria) | Open or laparoscopic prostatectomy | 40 to 80 years | 72 | 71 | 143 |
| Aboalsoud *et al* (2021) | Breast cancer | Unilateral modified radical mastectomy | 40 to 65 years | 20 | 20 | 40 |
| Titon *et al* (2021) | Tumours of prostate, stomach, pancreas, bile ducts, breast, colon, lung, uterus, kidneys, retroperitoneum | Removal of tumour mass | >18 years old | 22 | 23 | 45 |

***Table S2a*** *Study characteristics of published trials*

|  | **Opioid-free anaesthesia** | | | **Opioid-based anaesthesia** | | |
| --- | --- | --- | --- | --- | --- | --- |
| **Authors (Year)** | **Induction** | **Maintenance** | **Loco-regional analgesia** | **Induction** | **Maintenance** | **Loco-regional analgesia** |
| Rangel *et al* (2021) | Propofol  Dextroketamine Lidocaine Cisatracurium | Propofol Dextroketamine Lidocaine Dexmedetomidine | Transversus abdominis plane block with ropivacaine | Propofol  Dextroketamine Lidocaine Cisatracurium  **Fentanyl** | Propofol Dextroketamine Lidocaine Dexmedetomidine | Sham block (saline) |
| Aboalsoud *et al* (2021) | Propofol Atracurium | Isoflurane Atracurium Levobupivacaine | Thoracic paravertebral block with levobupivacaine | **Nalbuphine** Propofol Atracurium | Isoflurane Atracurium **Nalbuphine** | No |
| Titon *et al* (2021) | Propofol Lidocaine Magnesium sulphate Atracurium | Lidocaine Propofol Atracurium | Epidural (morphine) | Propofol Lidocaine Magnesium sulphate Atracurium  **Fentanyl** | Lidocaine Propofol Atracurium | Sham epidural (saline) |

***Table S2b*** *Anaesthesia regimes for published randomised controlled trials. Opioids are highlighted in bold.*

| **Full Title** | **Status of trial** | **Type of cancer** | **Surgical procedure** | **Population age eligibility** | **Number of participants** |
| --- | --- | --- | --- | --- | --- |
| Opioid Free Anesthesia in Breast Cancer Surgery: A Prospective Randomized Study  **(Trial 1)** | Recruiting | Breast cancer  TNM I or II | Radical mastectomy | 20 to 75 years | Estimated: 78 |
| To compare the efficacy of opioid free general anesthesia with opioid based general anesthesia on post operative morphine consumption in patients undergoing breast cancer surgery: a prospective randomized control study  **(Trial 2)** | Recruiting | Breast cancer | No details provided | 18 to 70 years | Target: 100 |
| Effect of a Perioperative Opioid Free Anaesthesia-Analgesia (OFA-A) Strategy on Surgical Stress Response and Immunomodulation in Elective VATS Lobectomy for NSCLC Lung Cancer: A Prospective Randomized Study  **(Trial 3)** | Recruiting | Non-small cell lung cancer early stage (up to T3N1M0) | Elective VATS lobectomy | 18 to 80 years | Estimated: 70 |
| The Efficacy and Safety of Opioid-free Anesthesia (OFA) for Non-small-cell Lung Cancer Resection and Its Underlying Clinical Value: A Prospective Study  **(Trial 4)** | Recruiting | Non-small cell lung cancer | Resection of tumour | 18 to 80 years | Estimated: 60 |
| Variation of the Neutrophil to Lymphocyte Ratio During Opioid-Free General Anesthesia Associated With Thoracic Wall Blocks Vs General Anesthesia, in Breast Cancer Quadrantectomy: a Randomized Controlled Trial  **(Trial 5)** | Recruitment status unknown | Breast cancer | Quadrantectomy | ≥18 years | Estimated: 68 |

***Table S3a*** *Study characteristics for ongoing randomised controlled trials*

|  | **Opioid-free anaesthesia** | | | **Opioid-based anaesthesia** | | |
| --- | --- | --- | --- | --- | --- | --- |
|  | **Induction** | **Maintenance** | **Loco-regional analgesia** | **Induction** | **Maintenance** | **Loco-regional analgesia** |
| **Trial 1**  Breast cancer | Propofol Lidocaine  Muscle relaxant | No description provided | Paravertebral block with ropivacaine | Propofol **Sufentanil** Muscle relaxant | No description provided | Sham block (lidocaine) |
| **Trial 2**  Breast cancer | No details Dexmedetomidine prior to induction | No details  Dexmedetomidine Magnesium sulfate after induction +/- Ketamine | Erector spinae block | No details **Fentanyl** prior to induction | No details  **Fentanyl** | No |
| **Trial 3**  Non-small cell lung cancer | Dexmedetomidine Lidocaine Propofol Ketamine Hyoscine Cisatracurium or rocuronium Magnesium sulphate Dexamethasone | Desflurane Dexmedetomidine Lidocaine Ketamine Paracetamol +/- dexketoprofen trometamol Ondansetron or droperidol | Wound infiltration with ropivacaine | Propofol **Fentanyl** Cisatracurium or rocuronium | Desflurane **Morphine Fentanyl** Paracetamol +/- dexketoprofen trometamol Ondansetron or droperidol | Wound infiltration with ropivacaine |
| **Trial 4**  Non-small cell lung cancer | No details of protocol Intravenous local anaesthetics | | Thoracic epidural | No details of protocol Minimal **sufentanil** and **remifentanil** | | Epidural **hydromorphone** |
| **Trial 5**  Breast cancer | Propofol Rocuronium | Propofol **+/- Fentanyl** | PEC I and serratus plane block | Propofol **Fentanyl** Rocuronium | Same as intervention group | No |

***Table S3b*** *Anaesthesia regimes for ongoing clinical trials. Opioids are highlighted in bold.*

| **Immunological parameter** | **Description** |
| --- | --- |
| Neutrophil-to-lymphocyte ratio | Neutrophils: either promote or inhibit cancer development and growth depending on the tumour microenvironment (Xiong *et al,* 2021)  Lymphocytes: key components of the adaptive immune system |
| Platelet-to-lymphocyte ratio | Platelets: facilitate metastatic spread through production of adhesion proteins, clotting factors, and interaction with both tumour and stromal cells (Lucotti and Muschel, 2020) |
| Lymphocyte-to-monocyte ratio | Monocytes: innate immune cells which can have both pro-tumour and anti-tumour effects |
| IL-4 | Anti-inflammatory |
| IL-6 | Pro-inflammatory |
| IL-8 | Pro-inflammatory |
| IL-10 | Anti-inflammatory |
| IL-12 | Pro-inflammatory |
| IL-17A | Pro-inflammatory |
| TNF-α | Pro-inflammatory |
| Oxidative stress profile (lipid peroxidation status and antioxidant capacity of plasma) | Lipid peroxidation: oxidative degradation of lipids mediated by free radicals (which are produced during cell metabolism)  Products of lipid peroxidation can cause DNA damage  Antioxidant capacity of plasma: antioxidants act against free radicals to protect the cell against damage |
| C-reactive protein (CRP) | Non-specific marker of inflammation |
| Natural killer cells (NK) | Cytotoxic function  Secrete IFN-γ (pro-inflammatory) |
| T helper cells | CD4^+^ helper cells enhance function of CD8^+^ cells and macrophages |
| CD8^+^ T cells | Cytotoxic function |
| Caspase 3 | Marker of apoptosis |
| AVP (arginine vasopressin) | Ectopic production in lung cancer  Hyponatraemic effects |
| Cortisol | Glucocorticoid hormone with immunosuppressive effects |
| HIF-1α | Subunit of transcription factor HIF  Regulates transcription of genes involved in regulation of oxygen levels, which is related to cell viability and proliferation |
| Vascular endothelial growth factor (VEGF) | Growth factor promoting angiogenesis |
| NF-κB | Set of transcription factors important in regulation of inflammation, cell activation and proliferation |
| Advanced lung cancer inflammation index | Prognostic marker (survival) He *et al* (2015) |
| Systemic immune inflammation index | Product of neutrophil-to-lymphocyte ratio and platelet count  Prognostic marker (survival) Biswas *et al* (2020) |

***Table S4*** *Description of immunological parameters evaluated across studies included in the review* [51]. *Citations are provided where information were obtained from another source*

|  | **Immunological** | | **Oncological** | |
| --- | --- | --- | --- | --- |
| **Authors (Year)** | **Parameters** | **Main Findings** | **Outcomes** | **Main Findings** |
| Rangel *et al* (2021) | Neutrophil-to-lymphocyte ratio | - No association between preoperative neutrophil-to-lymphocyte ratio and biochemical recurrence - Postoperative neutrophil-to-lymphocyte median rates not significantly different between groups | Biochemical recurrence  Biochemical recurrence-free survival | No significant differences in either outcome between groups |
| Aboalsoud *et al* (2021) | IL-10 TNF-α caspase 3 | ***OFA vs OBA:***   - Significant increase in IL-10 and caspase 3 at 24h and 7 days - Significant decrease in TNF-α after 24h and 7 days - Positive correlation between caspase 3 and IL-10 24hrs postoperatively in OFA and OBA | N/A | N/A |
| Titon *et al* (2021) | IL-4  IL-12 IL-17A TNF-α oxidative stress profile (lipid peroxidation status, antioxidant capacity of plasma) | ***OFA vs OBA:***   - Significant decrease in lipid peroxidation in postoperative period and significant increase in antioxidant capacity of plasma - No variation in IL-4, IL-17A and TNF-α - Propofol, fentanyl, lidocaine and magnesium sulphate all resulted in reduced IL-12 levels | N/A | N/A |

***Table S5*** *Main findings of published randomised controlled trials, comparing opioid-free anaesthesia to opioid-based anaesthesia*

*Appendix B – Search Strategies*

**Database: Cochrane Central Register of Controlled Trials (CENTRAL)**

**Date of search: February 8, 2022**

**Results:** **11**

| **Search term number** | **Search term** | **Results** |
| --- | --- | --- |
| #1 | opioid-free anaesthesia | 188 |
| #2 | cancer surgery | 48755 |
| #3 | #1 AND #2 | 32 |
| #4 | immune system | 10149 |
| #5 | inflammation | 48211 |
| #6 | neutrophil lymphocyte ratio | 750 |
| #7 | cytokine | 12327 |
| #8 | metastasis | 22517 |
| #9 | overall survival | 57941 |
| #10 | recurrence free survival | 13926 |
| #11 | angiogenesis | 4411 |
| #12 | tumour growth | 8582 |
| #13 | apoptosis | 4229 |
| #14 | immune response | 19715 |
| #15 | NK cell | 2267 |
| #16 | lymphocyte | 18487 |
| #17 | macrophage | 3845 |
| #18 | tumour associated neutrophil | 449 |
| #19 | platelet lymphocyte ratio | 265 |
| #20 | #4 OR #5 OR #6 OR #7 OR #8 OR #9 OR #10 OR #11 OR #12 OR #13 OR #14 OR #15 OR #16 OR #17 OR #18 OR #19 | 164313 |
| #21 | #3 AND #20 | 12 |
| #22 | Randomised controlled trials | 1251813 |
| #23 | #21 AND #22 | 11 |

**Database: Embase (Ovid)** (1974 – 2022 Week 5)

**Date of search: February 8, 2022**

**Results: 5**

| **Search term number** | **Search term** | **Results** |
| --- | --- | --- |
| 1 | Opioid-free.tw | 607 |
| 2 | Exp anaesthesia | 415378 |
| 3 | Cancer surgery | 336482 |
| 4 | Exp immune system | 2516004 |
| 5 | Exp inflammation | 4094891 |
| 6 | 1 AND 2 AND 3 | 39 |
| 7 | Exp neutrophil lymphocyte ratio | 14470 |
| 8 | Exp cytokine | 1709500 |
| 9 | Exp metastasis | 742654 |
| 10 | Exp overall survival | 391047 |
| 11 | Exp recurrence free survival | 42290 |
| 12 | Exp angiogenesis | 137166 |
| 13 | Exp tumour growth | 155637 |
| 14 | Exp apoptosis | 585132 |
| 15 | Exp immune response | 596846 |
| 16 | Exp natural killer cell | 85968 |
| 17 | Exp lymphocyte | 957263 |
| 18 | Exp macrophage | 363672 |
| 19 | 4 OR 5 OR 6 OR 7 OR 8 OR 9 OR 10 OR 11 OR 12 OR 13 OR 14 OR 15 OR 16 OR 17 OR 18 | 8110907 |
| 20 | 6 AND 19 | 13 |
| 21 | Limit 20 to (human and randomised controlled trial) | 5 |

**Database: MEDLINE/PubMed** (1946 – February 8, 2022)

**Date of search: February 8, 2022**

**Results: 2**

| **Search term number** | **Search term** | **Results** |
| --- | --- | --- |
| 1 | opioid free.tw. | 414 |
| 2 | exp Anesthesia/ | 200446 |
| 3 | exp Neoplasms/ | 3642951 |
| 4 | surgery.mp. or exp General Surgery/ | 2878398 |
| 5 | exp Immune System/ | 1226426 |
| 6 | exp Inflammation/ | 382039 |
| 7 | Neutrophil lymphocyte ratio.mp. | 3279 |
| 8 | exp Cytokines/ | 755529 |
| 9 | metastasis.mp. | 403550 |
| 10 | overall survival.mp. | 203608 |
| 11 | angiogenesis.mp. | 127521 |
| 12 | tumor growth.mp. | 92376 |
| 13 | exp Apoptosis/ | 308488 |
| 14 | Immune response.mp. | 182846 |
| 15 | natural killer cell.mp. | 11735 |
| 16 | exp Lymphocytes/ | 554194 |
| 17 | exp Neutrophils/ | 93197 |
| 18 | Platelet lymphocyte ratio.mp. | 1168 |
| 19 | 1 and 2 and 3 | 10 |
| 20 | 4 or 5 or 6 or 7 or 8 or 9 or 10 or 11 or 12 or 13 or 14 or 15 or 16 or 17 or 18 | 5529176 |
| 21 | 19 and 20 | 10 |
| 22 | Limit to (humans and randomised controlled trial) | 2 |

**Database: Web of Science**

**Date of search: February 8, 2022**

**Search terms:**

“opioid-free” (Topic)

AND

“anaesthesia” (Topic)

AND

“cancer surgery” (Topic)

**Refined by:**

Randomised

**Results: 2**

**Database:** **clinicaltrials.gov**

**Date of search: February 9, 2022**

**Search terms:**

“opioid-free anaesthesia”

AND

“surgery”

Condition/disease – cancer

**Results: 8**

*Appendix C – Additional study characteristics*

|  | **Age of participants** | | **Body Mass Index** | | **Gender (%)** | | **Tumour location** | |
| --- | --- | --- | --- | --- | --- | --- | --- | --- |
| **Authors (Year)** | **OFA** | **OBA** | **OFA** | **OBA** | **OFA** | **OBA** | **OFA** | **OBA** |
| Rangel *et al* (2021) | 67 (63-73)ᵃ | 67 (63-71)ᵃ | Normal: 34.2% Overweight: 39.7% Obese: 26% | Normal: 25.3% Overweight: 54.9% Obese: 19.7% | N/A | N/A | Prostate | |
| Aboalsoud *et al* (2021) | 58.9 ± 6.91ᵇ | 56.02 ± 6.12ᵇ | 27.15 ± 1.42ᵇ | 27.30 ± 1.49ᵇ | N/A | N/A | Breast | |
| Titon *et al* (2021) | <40: 3ᶜ 50: 9 60: 7 ≥70:3 | <40: 4ᶜ 50: 4 60: 11 ≥70: 4 | Underweight (BMI < 18.5): 4.76% Normal (18.5≥BMI≤24.9): 57.14% Overweight (25≥BMI≤29.9): 19.05% Obese (30≥BMI≤34.9): 14.26% Extremely Obese (BMI≥35): 4.76% | Underweight (BMI < 18.5): 9.09%  Normal (18.5≥BMI≤24.9):54.55%  Overweight (25≥BMI≤29.9): 22.73%  Obese (30≥BMI≤34.9): 4.55%  Extremely Obese (BMI≥35): 9.09% | Male: 59.09% Female: 40.91% | Male: 30.43% Female: 69.57% | Prostate: 28.57% Stomach: 14.29% Pancreas: 4.76% Bile ducts: 0.00% Breast: 4.76% Colon: 23.81% Lung: 4.76% Uterus: 9.52% Kidney: 4.76% Retroperitoneum: 4.76% | Prostate: 13.04% Stomach: 21.74% Pancreas: 0.00% Bile ducts: 8.70% Breast: 30.43% Colon: 4.35 Lung: 4.35% Uterus: 4.35% Kidney: 13.04% Retroperitoneum: 0.00% |

***Table S6a*** (above) *Additional study characteristics for published randomised controlled trials. Abbreviations:* ***OFA*** *– opioid-free anaesthesia;* ***OBA*** *– opioid-based anaesthesia*

- *a – median and interquartile ranges (25^th^ – 75^th^)*
- *b – mean and standard deviation*
- *c – absolute numbers*

*Appendix C – Additional study characteristics*

|  | **Smoking Status** | | **Duration of Surgery** | |
| --- | --- | --- | --- | --- |
| **Authors (Year)** | **OFA** | **OBA** | **OFA** | **OBA** |
| Rangel *et al* (2021) | No information provided | No information provided | No information provided | No information provided |
| Aboalsoud *et al* (2021) | No information provided | No information provided | 111.3 ± 22.12ᵇ | 100.5 ± 23.39ᵇ |
| Titon *et al* (2021) | Yes: 31.82% No: 68.18% | Yes: 26.09% No: 73.91% | Up to 60 minutes: 13.64% 60 to 120 minutes: 45.45% More than 120 minutes: 40.91% | Up to 60 minutes: 17.39% 60 to 120 minutes: 60.87% More than 120 minutes: 21.74% |

***Table S6b*** *Additional study characteristics for published randomised controlled trials. Abbreviations:* ***OFA*** *– opioid-free anaesthesia;* ***OBA*** *– opioid-based anaesthesia*

- *a – median and interquartile ranges (25^th^ – 75^th^)*
- *b – mean and standard deviation*
- *c – absolute numbers*
